# The Unconscious Mental Life of the Child

**Published:** 1916-03-25

**Authors:** 


### THE UNCONSCIOUS MENTAL LIFE OF THE CHILD.

### Platform of Child Study Society.

At the meeting of the Child Study Society on March 16, Dr. Ernest Jones delivered an address on "The Unconscious Mental Life of the Child." The chair was occupied by Mr. C. Holman, M.A. There were, said Dr. Jones, three critical questions which suggested themselves along such a line of study as that which he had adopted: (1) the extent to which they were justified in transferring to the normal conclusions based upon the abnormal; (2) the value of the examination of the adult mind as a basis for child study; (3) the reliance which could be placed on the psycho-analytic method of research. He dismissed the last of these with a mere mention. In regard to the first aspect, the differences between the working of a normal and of a neurotic mind were only those of degree: disease added no new element to the conditions present in the healthy mind, though the grouping of forces might be altered and the relative perspective modified. Indeed, such widespread incidence did neurotic tendencies reveal that it would be more correct to regard the absolutely healthy mind as abnormal.

### Is the Child Father to the Man?

In reference to the second question, the familiar saying that the child is father to the man was taken seriously by the psycho-analyst. The infantile mind still produced external manifestations in adult life, in a primitive form. The unchanged form of the infantile mind was buried in the depth of the unconscious, constituting the core and characteristic of the unconscious mind. "Unconscious" did not mean that no quality of consciousness attached to the mental process so termed, but that the conscious *ego* was unaware of it. Such ideas might accelerate activities without penetrating consciousness, such as in dream-life, and in slips of tongue and pen. In the psycho-analytic sense the expression "unconscious" meant processes which were unconscious with the reserve just mentioned: (2) dynamically active in the production of external manifestations; (3) of high significance to the personality; (4) endowed with certain mental attributes which sharply distinguished the processes from the familiar type of mental function. He believed that the extent to which the resistance of the child's mind operated had not yet been comprehended, and when a fond mother said her child told her everything, she was under an illusion: the child was both unable and unwilling to tell everything, because he could not formulate many of his innermost thoughts. From the beginning the child was protected from painful stimuli by the immediate gratification of his wants, but there were two clearly-marked stages in the mental development. The first was illusory, the second actual. These two systems were always at work: they were transmitted into the conscious and unconscious sections of the mind in later life, the first system remaining practically unaltered. The control of the primary system of thought by the secondary remained imperfect throughout life.

The Chairman traversed some of the statements made by Dr. Jones, and expressed the belief that the child did not of set purpose hold back its thoughts, but that it did not know anything about the matter: it lived in a world of fantasy, and the ideas therefrom took hold of the child. He also did not think the word "sexual" could be rightly applied until the occurrence of puberty: until then it was merely potential, in the sense that an acorn was a potential oak-tree.

### The Nerve Troubles of Later Life.

Dr. Jones replied. He said he did not think reason, or intellect, or the spiritual or ethical processes could penetrate to the unconscious at all: all we did was to modify the manifestations of the unconscious when they became accessible: when they came towards the conscious, they were capable of being acted upon by the impulses mentioned. Educational psychologists were doing much harm which could be avoided if only a few simple principles were employed based on the work of medical psychology. The nerve troubles of later life—the quarrels, frictions, incapacities, sense of inferiority—were all due to badly directed repression in early life. It was necessary to take the impulses present in the child's mind not at their adult value, but at the value placed upon them by the child. It was not right to say a child had such-and-such tendencies which must be squashed, but to ask how best could those tendencies, and the energy implied, be best directed to useful social purposes.

